# Quit Methods Used by US Adult Cigarette Smokers, 2014–2016

**DOI:** 10.5888/pcd14.160600

**Published:** 2017-04-13

**Authors:** Ralph S. Caraballo, Paul R. Shafer, Deesha Patel, Kevin C. Davis, Timothy A. McAfee

**Affiliations:** 1 National Center for Chronic Disease Prevention and Health Promotion, Office on Smoking and Health, Centers for Disease Control and Prevention, Atlanta, Georgia; 2 Center for Health Policy Science and Tobacco Research, RTI International, Research Triangle Park, North Carolina; 3 Department of Health Policy and Management, Gillings School of Global Public Health, University of North Carolina at Chapel Hill, Chapel Hill, North Carolina; 4 National Center for HIV/AIDS, Viral Hepatitis, STD, and TB Prevention, Division of HIV/AIDS Prevention-Intervention Research and Support, Centers for Disease Control and Prevention, Atlanta, Georgia

## Abstract

To quantify the prevalence of 10 quit methods commonly used by adult cigarette smokers, we used data from a nationally representative longitudinal (2014–2016) online survey of US adult cigarette smokers (n = 15,943). Overall, 74.7% of adult current cigarette smokers used multiple quit methods during their most recent quit attempt. Giving up cigarettes all at once (65.3%) and reducing the number of cigarettes smoked (62.0%) were the most prevalent methods. Substituting some cigarettes with e-cigarettes was used by a greater percentage of smokers than the nicotine patch, nicotine gum, or other cessation aids approved by the US Food and Drug Administration. Further research into the effectiveness of e-cigarettes as a cessation aid is warranted.

## Objective

Quitting cigarette smoking greatly reduces the risk of developing smoking-related diseases; although the health benefits are greater for people who stop at earlier ages, there are benefits at any age (1). The use of electronic cigarettes (e-cigarettes) has increased in the United States (2). Little is known about how the rise in e-cigarette use, particularly among current and former adult cigarette smokers, may have affected quitting behaviors. This study assessed common methods used to try to quit cigarettes among a nationally representative online sample of US adult current smokers surveyed from April 2014 through June 2016.

## Methods

We used data from a nationally representative longitudinal online survey of adult cigarette smokers in the United States. Survey participants were recruited from a probability sample of residential mailing addresses derived from the US Postal Service’s Delivery Sequence File, covering approximately 95% of all US households. Study invitation letters, which contained a website link and password to the selected household’s survey, were mailed to all sampled households. Each sampled household had a known probability of selection, and individual participants could not volunteer for study enrollment. All current smokers who participated at baseline were re-contacted for follow-up in the 5 waves that followed. Details on survey methods are available elsewhere (3). Free and informed consent of participants was obtained, and study methods were approved by the RTI International institutional review board.

The survey was conducted in 6 waves, from April 7, 2014, through June 2, 2016. Our analysis was based on 15,943 current cigarette smokers who reported having made at least one quit attempt in the previous 3 months. The data were weighted to reflect national distributions of sex, age, race/ethnicity, and education among cigarette smokers. Current cigarette smokers were defined as adults aged 18 years or older who had smoked at least 100 cigarettes in their lifetime and currently smoked “every day” or “some days.” A cigarette smoking quit attempt in the previous 3 months at follow-up was assessed by asking current smokers, “During the past 3 months, how many times have you stopped smoking for one day or longer because you were trying to quit smoking cigarettes for good?” Those who answered they tried to quit one or more times were categorized as having made a quit attempt and were subsequently asked, “When you last tried to quit smoking, did you do any of the following?” The survey provided a list of 10 quit methods, and respondents were asked to indicate which methods they used by responding yes or no to each method. Respondents were permitted to select multiple quit methods. We estimated the prevalence of using each quit method by calculating the weighted mean. We estimated 1) the prevalence of any quit method used (alone or in combination with any of the other 9 quit methods) and 2) the prevalence of using a single quit method among respondents who used only one method. All analyses were conducted using Stata version 13 (StataCorp LP).

## Results

Among participants who were invited and eligible to participate in this study, 50.2% completed the follow-up surveys. Most respondents in the study were male (51.3%) and non-Hispanic white (59.9%). The greatest proportion, by age, was aged 35 to 54 years (36.9%); by education, had graduated from high school (37.3%), and by income, had an annual household income of $20,000 to $49,999 (32.8%).

Overall, 74.7% of adult current cigarette smokers used multiple quit methods during their most recent quit attempt (Table 1). Giving up cigarettes all at once (65.3%) and gradually cutting back on cigarettes (62.0%) were the most common quit methods, followed by substituting some cigarettes with e-cigarettes (35.3%), using a nicotine patch or gum (25.4%), switching completely from cigarettes to e-cigarettes (24.7%), and switching from “regular” cigarettes to “mild” cigarettes (20.4%). Quit methods used less often were getting help from a doctor or other health professional (15.2%), using smoking cessation medications approved by the US Food and Drug Administration (FDA) (12.2%), and getting help from a website (7.1%) or a telephone quitline (5.4%).

**Table 1:** Prevalence of Any Quit Method Used (Alone or in Combination With Any of the Other 9 Quit Methods^a^) During the Most Recent Quit Attempt Among US Adult Cigarette Smokers Who Tried to Quit in the Previous 3 Months (n = 15,943), 2014–2016

Quit Method Used^a^	No. (Weighted %^b^)
**Reported using multiple quit methods**	12,417 (74.7)
Gave up cigarettes all at once	10,631 (65.3)
Gradually cut back on cigarettes	9,682 (62.0)
Substituted some regular cigarettes with e-cigarettes	5,861 (35.3)
Used nicotine patch or nicotine gum	4,047 (25.4)
Switched completely to e-cigarettes	3,721 (24.7)
Switched to “mild” cigarettes	3,376 (20.4)
Got help from a doctor or other health professional	2,963 (15.2)
Used FDA-approved medications such as Zyban or Chantix	2,374 (12.2)
Got help from a website such as Smokefree.gov	1,146 (7.1)
Got help from a telephone quitline	853 (5.4)

Abbreviation: FDA, US Food and Drug Administration.

a Respondent was permitted to select one or more of the 10 quit-method categories used in his or her most recent quit attempt; thus, categories are not mutually exclusive. Estimates are for those who used 2 or more quit methods (combined) and those who used only one.

b Percentages were based on a denominator of 15,943 and were weighted to reflect national distributions of sex, age, race/ethnicity, and education among cigarette smokers.

Among those who tried to quit in the previous 3 months, 25.3% reported using only one method to quit cigarettes during their most recent quit attempt (Table 2). The single most common quit method used alone was giving up cigarettes all at once (14.7%), followed by gradually cutting back on cigarettes (6.6%). Substituting some cigarettes with e-cigarettes or switching completely from cigarettes to e-cigarettes was each used by 1.1% of smokers. All other quit methods were used as a single quit method by less than 1% of smokers.

**Table 2:** Prevalence of Using Only One Quit Method During the Most Recent Quit Attempt Among US Adult Cigarette Smokers Who Tried to Quit in the Previous 3 Months (n = 15,943), 2014–2016

Quit Method Used^a^	No. (Weighted %^b^)
**Reported using only one quit method**	3,526 (25.3)
Gave up cigarettes all at once	2,040 (14.7)
Gradually cut back on cigarettes	905 (6.6)
Substituted some regular cigarettes with e-cigarettes	159 (1.1)
Switched completely to e-cigarettes	136 (1.1)
Used nicotine patch or nicotine gum	159 (0.8)
Used FDA-approved medications such as Zyban or Chantix	69 (0.4)
Switched to “mild” cigarettes	33 (0.3)
Got help from a doctor or other health professional	16 (0.2)
Got help from a website such as Smokefree.gov	6 (<0.1)
Got help from a telephone quitline	3 (<0.1)

Abbreviation: FDA, US Food and Drug Administration.

a Quit-method categories are mutually exclusive.

b Percentages were based on a denominator of 15,943 and were weighted to reflect national distributions of sex, age, race/ethnicity, and education among cigarette smokers.

## Discussion

Our study showed that most (74.7%) US adult smokers who tried to quit did so by using multiple quit methods as part of their most recent quit attempt. Giving up cigarettes all at once (“cold turkey”) and gradually cutting back on cigarettes continue to be the most commonly used methods (4,5). Before this study, we knew that some cigarette smokers were using e-cigarettes to attempt to quit (6–8), but we did not know how the rise in e-cigarette use, particularly among current adult cigarette smokers, may have affected quitting behaviors. We found that substituting some cigarettes with e-cigarettes was used by a greater percentage of smokers than the nicotine patch, nicotine gum, or other FDA-approved cessation aids. There is no conclusive scientific evidence that e-cigarettes are effective for long-term cessation of cigarette smoking (6). E-cigarettes are not approved by the FDA as a smoking cessation aid (9,10). FDA-approved medications have helped smokers to quit, in many instances doubling the likelihood of success (11). Finally, we found that most smokers who are switching to e-cigarettes or “mild” cigarettes are not switching completely. These smokers are not stopping their cigarette smoking.

This study has some limitations. Respondents to our online survey may have systematically different quitting habits than smokers nationally. However, the potential for bias was reduced through probability-based sampling, lack of opt-in to the panel, and weighting the sample to the US smoker population. Second, respondents were asked only to self-report the quit methods used as part of the most recent quit attempt in the previous 3 months; therefore, our estimates could be subject to recall bias and may not reflect the full breadth of quit methods used as part of a quit attempt during the study period.

Given that our data show that e-cigarettes are more commonly used for quit attempts than FDA-approved medications, further research is warranted on the safety and effectiveness of using e-cigarettes to quit smoking.

## References

[1] US Department of Health and Human Services. The health consequences of smoking — 50 years of progress: a report of the Surgeon General. Atlanta (GA): US Department of Health and Human Services, Centers for Disease Control and Prevention, National Center for Chronic Disease Prevention and Health Promotion, Office on Smoking and Health; 2014.

[2] US Department of Health and Human Services. E-cigarette use among youth and young adults: a report of the Surgeon General — executive summary. Atlanta (GA): US Department of Health and Human Services, Centers for Disease Control and Prevention, National Center for Chronic Disease Prevention and Health Promotion, Office on Smoking and Health; 2016.

[3] GfK. KnowledgePanel recruitment and sample survey methodologies. https://www.gfk.com/fileadmin/user_upload/dyna_content/US/documents/KnowledgePanel_Methodology.pdf. Accessed March 20, 2017.

[4] Chapman S , MacKenzie R . The global research neglect of unassisted smoking cessation: causes and consequences. PLoS Med 2010;7(2):e1000216. 10.1371/journal.pmed.1000216 20161722 PMC2817714

[5] Chapman S , Wakefield MA . Large-scale unassisted smoking cessation over 50 years: lessons from history for endgame planning in tobacco control. Tob Control 2013;22(Suppl 1):i33–5. 10.1136/tobaccocontrol-2012-050767 23591504 PMC3632984

[6] Hartmann-Boyce J , McRobbie H , Bullen C , Begh R , Stead LF , Hajek P . Electronic cigarettes for smoking cessation. Cochrane Database Syst Rev 2016;9(9):CD010216. 27622384 10.1002/14651858.CD010216.pub3 PMC6457845

[7] Bullen C . Electronic cigarettes for smoking cessation. Curr Cardiol Rep 2014;16(11):538. 10.1007/s11886-014-0538-8 25303892

[8] Biener L , Hargraves JL . A longitudinal study of electronic cigarette use among a population-based sample of adult smokers: association with smoking cessation and motivation to quit. Nicotine Tob Res 2015;17(2):127–33. 10.1093/ntr/ntu200 25301815 PMC4375383

[9] US Food and Drug Administration. FDA 101: smoking cessation products. http://www.fda.gov/forconsumers/consumerupdates/ucm198176.htm. Accessed March 20, 2017.

[10] US Food and Drug Administration. FDA warns of health risks posed by e-cigarettes. http://www.fda.gov/ForConsumers/ConsumerUpdates/ucm173401.htm. Accessed March 20, 2017.

[11] Fiore MC , Jaén CR , Baker TB , Bailey WC , Benowitz NL , Curry SJ , Treating tobacco use and dependence: 2008 update. Clinical practice guideline. Rockville (MD): US Department of Health and Human Services, Public Health Service; 2008.

